# A Longitudinal Pathway for Clinical Educators: a Case Study

**DOI:** 10.15694/mep.2018.0000129.1

**Published:** 2018-06-13

**Authors:** Veena Rodrigues, Emily Player, Peter Brooke, Hannah Massey

**Affiliations:** 1 Norwich Medical School

**Keywords:** medical education, career development, educational resaerch

## Abstract

This article was migrated. The article was marked as recommended.

It is increasingly being recognised that medical education research and scholarship are essential if we are to provide innovative, research-informed teaching to medical students. Developing a discrete pathway for the professional development of clinical educators side by side with clinical training would enable such training to be offered to high caliber candidates. However, the challenges faced by clinical educators trying to deliver both the clinical and educational components of their training to a high standard must not be underestimated. In addition, employing institutions need to consider and agree criteria for career progression of these clinical academics. Identifying a mentor who can guide professional development is crucial, particularly in the early stages.

## Background

The National Institute for Health Research (NIHR) was established in the UK in 2006 to transform research in the National Health Service (NHS) ( NIHR, 2016). One of its initiatives includes the opportunity for postgraduate medical trainees to combine academic training with their clinical specialty training through NIHR Academic Clinical Fellowships (ACF) and Clinical Lectureships (CLs). Typically, ACFs spend 75% of their time undertaking clinical training and 25% undertaking research or educationalist training. ACF posts are usually of a three year duration (four years for general practice trainees) during which the trainees are expected to develop their academic skills and prepare an application for a Fellowship to undertake a higher research degree or an application for a place on an educational programme (leading to a higher degree). Obtaining a fellowship to undertake a PhD degree at the end of the three-year post, or after completing their specialty clinical training is seen as a successful outcome.

Clinical Lectureships (CLs) allow trainees to undertake 50% research and 50% clinical training over four years. Academic trainees are recruited nationally through open competition. In addition to NIHR ACF posts, there are a small number of locally funded ACF posts to boost recruitment and retention of high caliber trainees to specific geographical regions and/or specialties.

## Rationale for ACFs in medical education

While clinical academics undertaking research linked to their clinical specialty have traditionally formed the majority of NIHR ACF/CL posts, clinical education training within these career pathways is a newer development. It signifies the recognition of the need for high quality educational research and scholarship underpinned by educational theory, as well as the need for contemporary, research-informed pedagogies to stimulate and facilitate lifelong learning among health professionals. NIHR funding in relation to high quality medical education research was revised to reflect the difficulty in ‘ *having potential for benefiting patients and the public within 5 years of its completion*’, by acknowledging that the research should ‘ *have the potential to have practical application.*’ ( NIHR, 2015).

Clinical education research, scholarship and innovation are essential for training future generations of health professionals. The notion of pathways for professional development of medical or clinical educators is not new, nor is it limited to the UK. Several authors have also discussed the need for measures to promote academic scholarship and career progression of clinical educators ( Castiglioni, 2013; Fischer, 2016; Levinson, 2000).

## Medical Education and Clinical Academic Training at the University of East Anglia (UEA), UK

The medical education department at UEA comprises a group of about 40 academics from a range of medical specialties, bio-sciences and social sciences. Individuals are involved in discipline-specific research and in medical education, technology enhanced learning and innovation, and other pedagogical developments. Several members are involved in progressing discourse at the national/ international level in their respective fields.

The UEA MBBS course produced its first graduates in 2007 (UEA, 2007). Since then, in the annual survey conducted by the General Medical Council (GMC), graduates of the UEA consistently rank among the most prepared for practice in the UK (GMC ref). Four areas of good practice were identified in a GMC quality assurance visit in 2015: the Medical Aspirations Programme, Interprofessional Learning, Assessment of Professionalism, and Remediation of final year medical students.

Staff development and mentoring is a key activity of the department and members mentor junior educators in preparation for their future roles as clinical and educational supervisors and/ or tutors. Several Faculty are Members, Fellows and/ or Senior Fellows of the Higher Education Academy and the Academy of Medical Educators. Departmental activities focus on evidence-informed teaching, enterprise, innovation and engagement activities, and medical education scholarship. The innovative approaches to medical education on the MBBS course and provision of opportunities for student-selected-study in medical education, and to undertake an intercalated degree in Clinical Education have translated into increased interest in training as clinical educators and undertaking medical education research and scholarship. Students are encouraged and supported to present their research at Regional, National and International events and to publish in peer-reviewed journals.

Within postgraduate education and faculty development, recent innovations in the department have included the development of massive open online courses on the FutureLearn platform to meet the development needs of clinical supervisors ( Rodrigues, 2016). Over 18,000 individuals from 148 countries have completed these courses to date. Feedback from learners suggests that the courses have met an universal learning need among clinicians to fulfil their role as supervisors, tutors or practice educators. The innovative curriculum development, design and delivery also meets the needs of the busy 21st century healthcare professional and stimulates interprofessional learning and reflection (Rodrigues, 2015).

The recent shortfall of medical students choosing GP training is likely to result in a severe strain on existing GPs. To meet the current need, 50% of all medical students need to specialise in GP, currently this percentage sits between 9-30% across all medical schools ( HEE 2016). Offering ACF opportunities could potentially stimulate recruitment into GP training and provide teaching opportunities, utilise the underused computer systems in primary care for research, and create effective mentors for future generations of medical students ( Campbell, 2015; Gray, 2018; Spooner, 2018). A similar strategy could potentially enhance recruitment and retention into other shortage specialties in the UK.

In 2017, three ACFs (two in Paediatrics, locally funded; one in General Practice, NIHR funded) joined the UEA department of medical education, putting postgraduate medical education in the spotlight for the first time within the East of England. As part of their training, the ACFs are enrolled onto the part-time Master of Clinical Education (MClinEd) degree at UEA. This postgraduate taught programme is accredited by the Higher Education Academy (HEA) for professional recognition as a Fellow of the HEA. The course covers educational theory, contemporary teaching, learning and assessment methods, curriculum development and evaluation, and leadership and faculty development. Learners also undergo training in qualitative and quantitative research methods, to inform and support development of the educational research project under expert supervision for the dissertation. The ultimate aim of the fellowship is to provide training and experience in educational research and scholarship, underpinned by educational theory. This would enable the ACFs to pursue a career as a clinical academic in their chosen specialty, with a focus on medical education.

We present three personal narratives to demonstrate the diversity of pathways through which junior doctors develop themselves as clinical educators.

## Personal Narratives

1) Dr Emily Player, Academic Clinical Fellow in General Practice and Medical Education

Specialty and interests: I am a GP ACF in medical education with special interest in online learning and medical humanities. I am enrolled on a part-time Master of Clinical Education course; in addition, I do some teaching for medical students in primary care.

Pathway into this role: I completed an academic foundation programme where I developed an interest in medical education. I was fortunate to be involved in creating some innovative online medical education courses on the FutureLearn platform ( Rodrigues, 2016). After completing the first year of GP training, I decided that I would like to continue my academic interests. I missed the balance that academic and clinical posts provided and found I was spending my spare time undertaking academic work but without a recognised role. I was fortunate in being able to switch my training and become an NIHR GP ACF in medical education

Benefits: The main benefits are the twofold opportunities - clinical and academic. As an ACF, I am completing a funded, part-time master’s degree and can use my academic time to attend and present at conferences, get involved in publications, and access opportunities to apply for research fellowships. Being surrounded by academic GPs has led me to consider this as a career pathway. The research and teaching has influenced my clinical practice, keeping my knowledge fresh and enhancing my clinical reasoning skills. Having the academic time has also enabled me to cope with the stresses of being a GP, and amplified my enthusiasm for the job.

Challenges: There are a few practical challenges such as completing my training portfolio, which is not designed for ACFs, enrolling on a master’s program and navigating the university systems. Good communication has been an ongoing necessity to make this role work. There are many clinical, administrative, and academic colleagues who are unsure of what I do, and where I fit into the ‘system’. Communicating this well and explaining time constraints has been crucial to minimise feeling overwhelmed by work, research, master’s study and teaching. The GP training scheme is relatively short, therefore fitting in my exams, ACF and fellowship application prior to CCT is difficult. Moreover, going on to do a PhD after CCT can feel uncomfortable as the early years after CCT are known to be a steep learning curve. Working in isolation can also be a challenge. I have overcome this by ensuring I have a presence both physically and socially in both environments, thus helping me feel like part of both teams.

2) Dr Peter Brooke, Academic Clinical Fellow in Paediatrics and Medical Education

Specialty and interests: I am a first year Paediatric trainee with an interest in neonatology, and teaching at undergraduate and postgraduate levels. My research interests include peri-viable counselling and Post Traumatic Stress Disorder (PTSD) in parents after a neonatal intensive care unit admission. My ACF time is spent flexibly, split between a Masters in Clinical Education, teaching, and research.

Pathway into this role: I had taught medical students for some time but had struggled to find time to pursue this while working full time. I had also wanted to gain a formal teaching qualification since medical school, but I was not keen to extend my training time of eight years even further by doing this. When the opportunity came up to do an ACF job without necessarily extending my training time, I jumped at the chance and was thus able to combine my interests of neonatology and teaching.

Benefits: There are a number of exciting benefits to being an ACF. From a personal academic perspective, I get time to do research, attend conferences and publish while still working full time. This can be a route into a PhD later in my training. The big advantage for me in this situation is that I don’t have to extend my training at a time in my life when I have a young family. I can get on with research without slowing down the march to Consultancy. My “real” job gets the benefit of my ever-improving teaching skills and training session too. This makes up for the small amount of chaos I cause on the rota.

From an educational perspective, the ACF job has been really rewarding too. I am enrolled on the part-time Master of Clinical Education course - this is a really tangible benefit to this 3 year block. Further, I slot in teaching when I can so that I can make theory-practice links.

Finally, from a personal point of view, having the freedom to flex my time slightly is a huge benefit. For instance, if I have an ACF day on which I have childcare responsibilities, I manage to catch up on ACF work at the weekend. Similarly, with the intensity of current Paediatric rotas, the quality of life improvement of having a busy on-call week broken up by a day of teaching cannot be underestimated.

Challenges: The biggest initial challenge I faced was determining exactly what my “job” was. I knew I wanted to take the Masters course, but beyond that, my research plans felt vague. The first few months were a significant learning curve of finding a supervisor, literature searches and trying to pin down a research question.

Time is probably the next biggest issue I continue to grapple with. As with most Education ACFs I have used my one day a week allowance to attend the Master’s course. However, combining my coursework, finishing my research protocol to submit for ethics approval, and delivering teaching sessions, that time scarcely feels enough. It has been a rapid lesson in how to say no to all kinds of interesting projects as there simply isn’t enough time to do everything. When discussing projects now I find myself thinking carefully about the benefit of a project and the time commitment required.

Although I have not struggled with this issue myself, I have spent at least some part of this year in fear of upsetting my rota coordinator! To go from a “normal” SHO rota slot, to having to find 1 day a week for mandatory ACF time in a tight and understaffed rota is a challenge. Early planning and coordination with the rota coordinator was crucial here as it allowed them to re-shuffle other junior doctors to cover my ACF days.

3) Dr Hannah Massey, Academic Clinical Fellow in Paediatrics and Medical Education

Specialty and interests: I am currently a Paediatric ST3. From an educational perspective, my research is focused on better integration of medical students into health care teams. This is becoming increasingly challenging with fragmented shift patterns and a growing number of medical students.

Pathway into this role: I would like to say I had a neatly mapped out career plan, but that would not be at all true. I had written off becoming an academic after a rather disastrous intercalated degree (as an undergraduate medical student) which seemed to involve endless pipetting. However, I had always enjoyed teaching and became a clinical supervisor at the University of Cambridge during my Foundation training. As part of this program, I received training on how to teach. This opportunity really opened my eyes to the huge amount of educational research that goes into training the next generation of doctors. It was also when I decided that I wanted to play a role in that research. I saw the ACF post advertised and loved the idea of being able to undertake educational research while also continuing my Paediatric training.

Benefits: The main benefit is the ability to brainwash all medical students into becoming Paediatricians. Aside from that, being able to undertake the part time Master of Clinical education course has allowed me to develop into a much more effective teacher. As well as learning how to teach, I have also learnt so much more about Paediatrics from the research and planning needed to deliver lectures.

Challenges: Being the first group of clinicians to undertake an educational ACF is daunting. There is no clear path to follow and it is challenging meeting the demands of different stakeholders. You often feel you are walking a tightrope trying to balance teaching and research commitments to keep everyone happy. This has led to excessive workloads at times. However, it has been a fantastic experience so far and I am looking forward to the next two years.

## Discussion

The personal narratives presented demonstrate the variety of points at which medical students and junior doctors begin to develop an interest in education and educational research.

**Figure 1.**
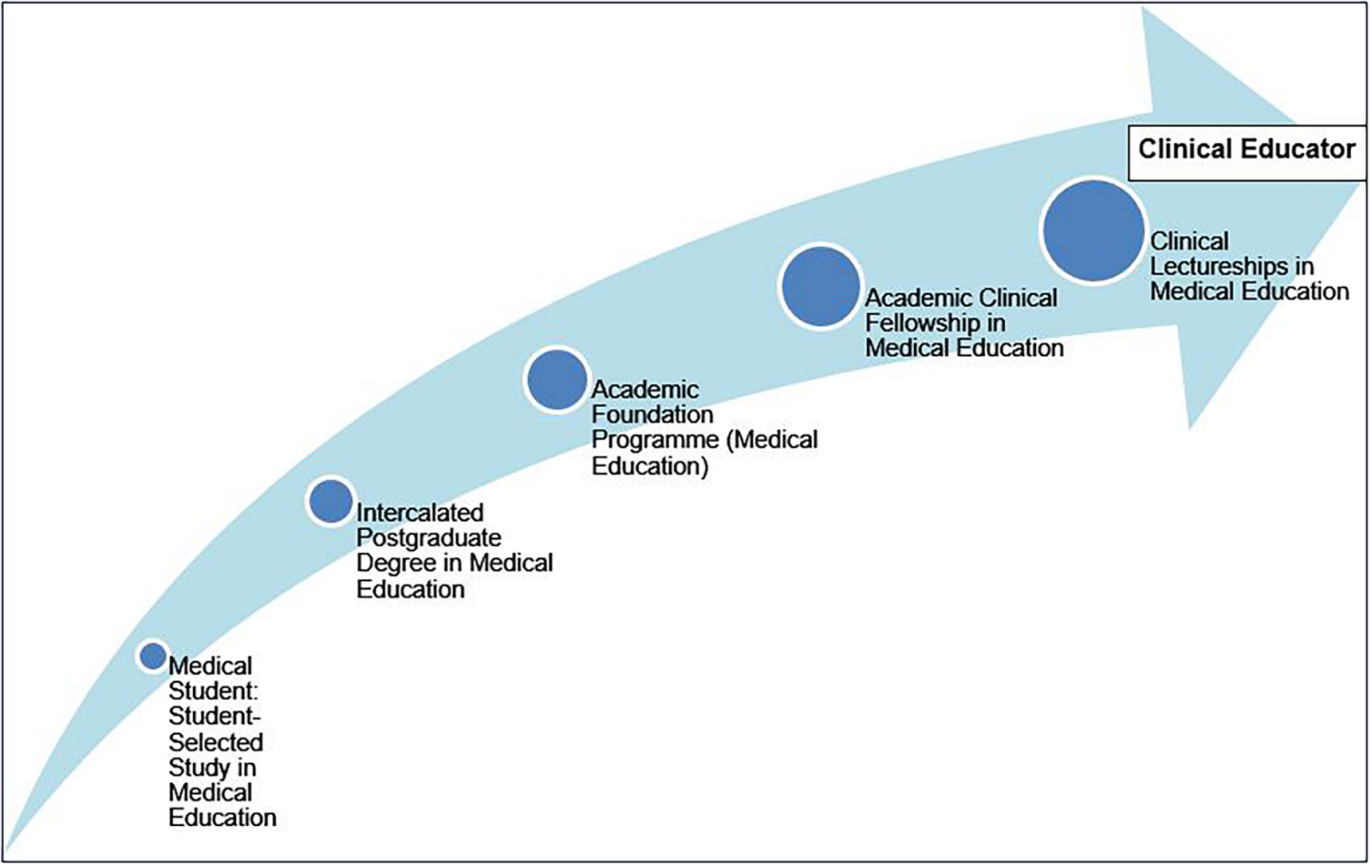
Pathway for Clinical Educator Development in the UK

For medical education departments and faculty, there are several opportunities to introduce medical education as a topic of interest early in undergraduate medical education through student selected studies, research skills development, teaching and presentation skills development, etc. Where intercalation to undertake postgraduate study is not mandatory, medical schools could encourage students to consider a Master’s programme in medical education or involvement in medical education research. Similarly, stimulating interest among medical students in undertaking academic foundation training, with a focus on medical education, could be the precursor to a career pathway as a clinical academic, with an interest in medical education. Within the East of England, several posts providing an opportunity to combine medical education with clinical training, including enrolment on a part-time Postgraduate Certificate in Medical Education, are now available within the Foundation training programme. With the funding of clinical fellowships and clinical lectureships in medical education by NIHR or local bodies, a distinct pathway for medical education scholarship and research and indeed development of clinical educators is now beginning to emerge ( Figure 1).

## Top Tips for Junior Doctors with an interest in Medical Education

**Figure:**
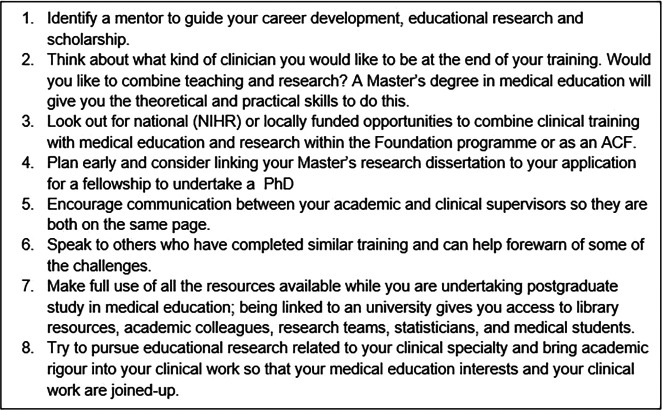


## Conclusion

NIHR clinical academic pathways for medical students and postgraduate doctors in the UK have broadened from clinical research for patient benefit to include high quality medical education research that has the potential to change educational practice. Awareness of this career pathway needs to be developed early on in medical schools with opportunities to study medical education as a student selected component, develop research and teaching skills, and undertake intercalated degrees in medical education. The availability of academic foundation programme posts, clinical fellowships and clinical lectureships in medical education provides increased opportunities to support the professional development, research and scholarship of future clinical educators.

## Take Home Messages

• Clinical academic pathways for medical students and postgraduate doctors in the UK have broadened to include medical education and research.
• Awareness of this career pathway needs to be developed early on in medical schools.
• A variety of opportunities are available to support the professional development, research and scholarship of future clinical educators.
• Identifying a good mentor is extremely helpful particularly in the early stages of your career.
• Innovative medical education cannot be delivered without high quality educational research.

## Notes On Contributors

Veena Rodrigues is a Professor of Public Health and Medical Education at Norwich Medical School, UEA, Norwich, UK. She is a Consultant in Public Health Medicine by background and Head of the Medical Education Department at Norwich Medical School. Professor Rodrigues’ areas of interest include technology enhanced learning, massive open online courses, curriculum development and delivery, and mentoring of junior educators.

Dr Emily Player is a GP ACF in Medical Education at Norwich Medical School, UEA, Norwich, UK with a special interest in online learning and medical humanities.

Dr Peter Brooke is a Paediatric ACF in Medical Education at Norwich Medical School, UEA, Norwich, UK with a special interest in neonatology, and teaching at undergraduate and postgraduate levels.

Dr Hannah Massey is a Paediatric ACF in Medical Education at Norwich Medical School, UEA, Norwich, UK with a special interest in teaching and integration of medical students into health care teams.
