# Supplementary material for: A Longitudinal Pathway for Clinical Educators: a Case Study
Source: MedEdPublish (2016). 2018 Jun 13;7:129. [Version 1] doi: 10.15694/mep.2018.0000129.1 (PMC10699366; doi:10.15694/mep.2018.0000129.1)
Supplement: Peer review history

# Reviewer report: Reviewer response for version 1

**DOI:** 10.21956/mep.19604.r29120

**Published:** 2018-07-13

**Authors:** Susan Van Schalkwyk

**Affiliations:** 1 Stellenbosch University

This review has been migrated. The reviewer awarded 3 stars out of 5 The focus of this case study is an important one, particularly in light of the growing numbers of clinicians who are choosing to enhance their teaching practice in more formal ways. The article highlights some of the challenges that these clinicians experience and suggests that creating a clear career trajectory for these clinicians would be of value. I very much liked the idea of progression, and the idea of the pathway resonated with me. I did, however, find the article to be very UK-centric and as a non-UK reader struggled to make sense of the different levels in terms of fellowships and the like, that were being described. The personal narratives were interesting, as was the focus on an early discussion about career and choices. Further work is needed in this area.

Reviewer Expertise:

NA

No decision status is available

# Reviewer report: Reviewer response for version 1

**DOI:** 10.21956/mep.19604.r29121

**Published:** 2018-06-18

**Authors:** P Ravi Shankar

**Affiliations:** 1 American International Medical University

This review has been migrated. The reviewer awarded 4 stars out of 5 This is an interesting article on a longitudinal pathway for clinical educators in the United Kingdom. The article is well written though I agree with the other reviewer that it is mainly focused on the United Kingdom perspective. I am happy that MedEdPublish offers authors a variety of formats for their manuscripts and I liked the personal stories of the three clinical educators. Training to become a doctor is a long process and a fellowship may provide an opportunity to doctors to acquire advanced degrees and fellowships in medical education. Balancing clinical work, teaching students and personal educational requirements can be tough as mentioned by the authors. Educational research is vital for continued development of medical education. This article will be of particular interest to clinicians interested in pursuing an academic medical career.

Reviewer Expertise:

NA

No decision status is available

# Reviewer report: Reviewer response for version 1

**DOI:** 10.21956/mep.19604.r29119

**Published:** 2018-06-14

**Authors:** Subha Ramani

**Affiliations:** 1 Harvard Medical School, Brigham and Women's Hospital

This review has been migrated. The reviewer awarded 3 stars out of 5 I enjoyed reading the personal narratives and appreciate the different pathways trainees and faculty level professionals can design their trajectory in training to be educationalists. I also read with great interest about the UK based fellowships which are recently being awarded to those engaging in medical education research. This is a welcome trend. I also agree with the authors that more countries, institutions and organizations need to prioritize educational research as an important field of research and promote the development of specialists in this arena.But many of the details of this fellowship and this structure are UK centric- at least at this time. Those interested in education careers outside of the UK can get some insights from this narrative, but need to explore opportunities in their own context.It is important for many countries around the world to offer clear career advancement opportunities for educators / educationalists.

Reviewer Expertise:

NA

No decision status is available
